# *Cutibacterium acnes* biofilm formation is influenced by bone microenvironment, implant surfaces and bacterial internalization

**DOI:** 10.1186/s12866-024-03422-1

**Published:** 2024-07-20

**Authors:** Jennifer Varin-Simon, Marius Colin, Frédéric Velard, Min Tang-Fichaux, Xavier Ohl, Céline Mongaret, Sophie C. Gangloff, Fany Reffuveille

**Affiliations:** 1 Université de Reims Champagne-Ardenne, BIOS, Reims, France; 2 Université de Reims Champagne-Ardenne, UFR Pharmacie, Reims, France; 3 Université de Reims Champagne-Ardenne, UFR Odontologie, Reims, France; 4 CHU Reims, Service d’Orthopédie et Traumatologie, Reims, France; 5 CHU Reims, Service Pharmacie, Reims, France

**Keywords:** *Cutibacterium acnes*, Biofilm matrix, Prosthesis joint infection, Internalization

## Abstract

**Background:**

The bacterial persistence, responsible for therapeutic failures, can arise from the biofilm formation, which possesses a high tolerance to antibiotics. This threat often occurs when a bone and joint infection is diagnosed after a prosthesis implantation. Understanding the biofilm mechanism is pivotal to enhance prosthesis joint infection (PJI) treatment and prevention. However, little is known on the characteristics of *Cutibacterium acnes* biofilm formation, whereas this species is frequently involved in prosthesis infections.

**Methods:**

In this study, we compared the biofilm formation of *C. acnes* PJI-related strains and non-PJI-related strains on plastic support and textured titanium alloy by (i) counting adherent and viable bacteria, (ii) confocal scanning electronic microscopy observations after biofilm matrix labeling and (iii) RT-qPCR experiments.

**Results:**

We highlighted material- and strain-dependent modifications of *C. acnes* biofilm. Non-PJI-related strains formed aggregates on both types of support but with different matrix compositions. While the proportion of polysaccharides signal was higher on plastic, the proportions of polysaccharides and proteins signals were more similar on titanium. The changes in biofilm composition for PJI-related strains was less noticeable. For all tested strains, biofilm formation-related genes were more expressed in biofilm formed on plastic that one formed on titanium. Moreover, the impact of *C. acnes* internalization in osteoblasts prior to biofilm development was also investigated. After internalization, one of the non-PJI-related strains biofilm characteristics were affected: (i) a lower quantity of adhered bacteria (80.3-fold decrease), (ii) an increase of polysaccharides signal in biofilm and (iii) an activation of biofilm gene expressions on textured titanium disk.

**Conclusion:**

Taken together, these results evidenced the versatility of *C. acnes* biofilm, depending on the support used, the bone environment and the strain.

**Supplementary Information:**

The online version contains supplementary material available at 10.1186/s12866-024-03422-1.

## Background

The constant evolution of biomaterials dedicated to prosthesis and 3D printing scaffolds responds to the increase in demand for implants of bone and joint prostheses due to population aging. However, arthroplasty procedures still face the additional risk factor of infection [1]. Prosthesis joint infections (PJI) occurs in 1–2% of primary arthroplasties and have become a major clinical issue [2]. *C. acnes*, an anaerobic Gram-positive and skin commensal bacterium known to be involved in acne, can be responsible for up to 10% of bacterial PJI [3, 4]. Its highest incidence was observed in shoulder arthroplasty (accounting for up to 50% of all *C. acnes* PJIs), with the hypothesis that *C. acnes* is more prone to naturally colonize the shoulder skin [5, 6]. The complexity of diagnosis in the case of *C. acnes*-related-PJI is often linked to the absence of clinical symptoms except a mechanical discomfort for the patient [6, 7]. One hypothesis raised to explain such a discrete manifestation is that *C. acnes* could escape the host immune system and antibiotic treatment due to internalization into host cells (i.e. osteoblast or mesenchymal stem cells), or thanks to biofilm formation [8–10]. The internalization of bacteria could affect some of their behaviors like the biofilm formation after bacterial release from the cells [8, 9]. Biofilm is a community of bacteria under different metabolic states, glued in an extracellular matrix essentially composed of proteins, extracellular DNA (eDNA) and exopolysaccharides [11, 12]. Moreover, bacteria embedded in biofilms have the capacity to resist to very high concentration of antibiotics (10 to 100 higher than minimal inhibition concentration, MIC, of planktonic counterparts in vitro), and to host immune system [7, 13]. *C. acnes* forms biofilm on different prosthesis-related materials like titanium alloys (TiA), cobalt-chromium-molybdenum (CoCrMo), polyether ether ketone (PEEK) and stainless steel used for hybrid prosthesis manufacturing [14, 15], but also polymethylmethacrylate bone cement [16]. Thus, the biofilm appears as a major clinical issue, causing a persistence of bacteria and inducing the necessity to treat patients with high doses of antibiotics during a period of several months, increasing the risk to select resistant bacteria [7].

Biofilm requires a precise comprehension to determine efficient treatments. However, little is known on biofilm characteristics of PJI-associated *C. acnes*. It has been shown that the biofilm matrix of *C. acnes* acne isolates consists of major components including eDNA and proteins. These components play important roles for bacteria attachment and biofilm maturation [17]. Additionally, glycolysis residues are present in *C. acnes* biofilm matrix, with a different from poly-N-acetylglucosamine found in *S. aureus* biofilm matrix [18, 19]. Transcriptomic and proteomic studies on different *C. acnes* strains have highlighted genes and proteins implicated in biofilm formation: amino-acid metabolism genes [18], replication and division genes, cell wall synthesis genes, stress-induced genes and strain-dependent virulence genes like Christie–Atkins–Munch–Petersen (CAMP) factor 4 and lysozyme M1 [20]. Both an *in* vitro biofilm study and a rabbit biofilm-infection model of *C. acnes* identified an over-production of some proteins like elongation proteins (*tuf* and *fusA*), glycolysis and tricarboxylic acid cycle (TCA) enzymes in the biofilms [19, 21].

While these results were obtained on *C. acnes* biofilms grown on different supports (plastic, cellulose acetate, glass beats after isolation of rabbit bone), few studies described the *C. acnes* biofilm on prosthesis-mimicking support to represent PJI context. Notably, environmental conditions and surfaces might strongly influence the biofilm structure and composition [22].

*C. acnes* infection mainly occurs in the presence of prosthesis material [23]. We hypothesize that the nature of implant material plays an important role in *C. acnes* biofilm development. The aim of this study was to show the need to develop more specific model for study PJIs. For that, non-PJI- and PJI-related *C. acnes* biofilm formation on plastic support, used in classic in vitro model, and textured titanium representative of bone prosthesis were characterized and compared using different methods. Moreover, the impact of *C. acnes* internalization by SaOS-2 cells on biofilm development was also investigated.

## Materials and methods

### Bacterial strains and culture

Clinical *C. acnes* strains were isolated and collected at the laboratory of bacteriology of Reims University Hospital Center (CHU Reims). Two clinical isolates were non-PJI-related strains (C2 and C5) and two other clinical strains were isolated from bone and prosthesis infection (PJI2 and PJI8). Clinical strains were registered on clinicaltrials.gov (NCT03950063). Their multi locus sequence typing (MLST) profiles, previously identified [9], are listed in Table 1.

**Table 1:** Multilocus sequence typing (MLST) profiles and phylotypes of non-PJI-related and PJI-related *C. acnes* strains

Clinical strain	Origin	Sequence Typing	Clonal Complex MLST	Phylotype
C2	Skin contamination	107	CC107	IC
C5	Skin contamination	1	CC1	IA1
PJI2	Shoulder prosthesis	1	CC1	IA1
PJI8	Shoulder prosthesis	152	CC5	IB

*C. acnes* was isolated on Columbia agar with 5% sheep blood (BioRad, Hercules, California, USA), under anaerobic conditions using the GenBox system (Biomerieux, Marcy l’Etoile, France), at 37 °C, for 5 days. For all assays, one colony from each *C. acnes* strains was added on 1.5 mL- tube previously filled with Brain Heart Infusion (BHI) broth (BioRad, Hercules, California, USA). After 5 days incubation at 37 °C, absorbance at 600 nm was adjusted to 1 and bacteria were seeded to a final dilution of 1/100 (or 1/10 for RNA extraction) in BHI or diluted BHI (dBHI: 90% sterile water and 10% BHI), in 24 well-plates with different supports and incubated under anaerobic conditions, at 37 °C, for 5 days, for each experiment. Two different media (BHI and dBHI) were tested to compare the classical lab condition (i.e. BHI) to the nutrient-limited condition (i.e. dBHI).

### Supports used

In this study, Thermanox™ plastic coverslip (1.3 cm², Thermo Fisher Scientific, Waltham, Massachusetts, USA), raw titanium alloy disks (diameter 12 mm x height 5 mm, 1.1 cm²) (ACNIS, Chassieu, France) and titanium alloy disks shot-peened by CRITT-MI (1.1 cm², Charleville-Mézières, France) were used. Titanium alloy disks (TiAl6V4) used in this study are in accordance with the medical standard ISO 5832-3 (or ASTM F136). The roughness was obtained by microbead projection on the surface of the titanium alloy disk. The difference between both raw and microblasting surface is in the micrometer range. The shot-peened titanium (also name textured titanium) disk obtained was the support chosen as prosthesis mimicking material. Raw titanium disk (with a smooth surface) was used to assess the effect of the texturation as a surface modification could increase bacterial adhesion and biofilm formation and composition. The plastic coverslip was chosen as the classic in vitro model (used as lab condition reference). An initial count was carried out to confirm that the same quantity of bacteria was deposited for each *C. acnes* strains, allowing their behavior to be compared (see Additional file 1 A).

### *C. acnes* adhesion

Supports were deposited in each well of a 24-wells plate. Then, one milliliter of the bacterial inoculum in BHI or dBHI prepared above (8 × 10^6^ to 1.1 × 10^7^ bacteria/mL) was added to each well. After 5 days anaerobic incubation, supports were immersed in BHI or dBHI to be washed and immediately transferred to a 15 mL tube containing 2 mL of BHI or dBHI to be incubated 5 min in ultrasonic bath (40 kHz) to detach biofilm-embedded bacteria. To quantify non-adherent bacteria, 100 µL were collected before ultrasonication and serial dilutions were done. To quantify live adherent and planktonic bacteria, 100 µL were collected after ultrasonication and serial dilutions were done. Then dilutions were seeded on blood agar plates using automatic seeder EasySpiral (Interscience, Saint-Nom-la-Bretèche, France). After 5 days anaerobic incubation, the number of recovered colony-forming units (CFU) was determined using automatic counter SCAN 1200 (Interscience, Saint-Nom-la-Bretèche, France) and the quantity of live adherent bacteria was determined as follows: CFU/mL (after) – CFUL/mL (before) and area normalized (per cm²). At least three independent experiments were performed with technical duplicate for each *C. acnes* strain.

### Composition of *C. acnes* biofilm extracellular components

After 5 days of incubation in dBHI, mimicking a nutrient-poor environment, under anaerobic conditions, supports were washed twice in Phosphate Buffered Saline (PBS) and stained with the different labelled solution, listed in Table 2 (Invitrogen, Waltham, Massachusetts, USA, for all fluorescent markers).

**Table 2:** Labels used for biofilm and matrix composition

	Labels	Concentration	Final Target
	SYTO9™	1 µM	Live bacteria
Solution 1	Propidium iodide (PI)	20 µM	Damaged and dead bacteria
	SYTO9™	1 µM	All bacteria
Solution 2	SYPRO Ruby^®^	Ready to use	Proteins
	SYTO9™	1 µM	All bacteria
Solution 3	Concanavalin A (conA) Alexa Fluor™ 350 conjugate	100 µg/mL	Simple polysaccharides like mannose
	SYTO9™	1 µM	All bacteria
Solution 4	Wheat Germ Agglutinin (WGA) Alexa Fluor™ 350 conjugate	100 µg/mL	Complex exopolysaccharides like PNAG
	TOTO-3™ iodide	2 µM	Extracellular DNA (eDNA)

Each solution was diluted in sterile NaCl 0.9%. After 30 min of incubation in the dark, at room temperature, each material was washed twice in PBS before being flipped upside-down and placed in a Krystal 24-well plate with glass bottom (Porvair, United Kingdoms). Biofilm was observed and imaged using confocal scanning laser microscopy (CSLM) LSM 710 NLO (ZEISS, Oberkochen, Germany). 3D-reconstruction were created, and fluorescence volumes were quantified, using IMARIS software. Three independent experiments were performed and at least two areas were captured for each condition for titanium disks and plastic coverslip. Controls without bacteria were realized for each labels solution to confirm that the different fluorochrome did not attach on plastic coverslip or titanium disks.

### mRNA expression of *C. acnes* genes

mRNA analysis was undertaken to evaluate the expression of genes implicated in stress response and biofilm formation, previously identified by Jahns AC. et al, 2016 and Achermann Y. et al., 2015 [20, 21]. Each *C. acnes* strains was seeded in stationary phase culture at a dilution of 1/10 in dBHI in the presence of different supports. The incubation lasted 48 h for planktonic and 5 days for biofilm under anaerobic atmosphere, at 37 °C, adapting from Jahns AC. et al., 2016 [20]. Planktonic bacteria were pelleted by centrifugation 8 min, at 5000 g, 4 °C. After 5 days of incubation, adherent bacteria embedded in biofilm were detached by ultrasonic bath (40 kHz) for 5 min, after two PBS washes and pelleted by centrifugation 8 min, at 5000 g, 4 °C. Pellets were conserved at -80 °C before RNA extraction. Total RNAs were extracted using MasterPure™ RNA Purification Kit (Lucigen, Middleton, Wisconsin, USA) according to the manufacturer’s protocol. Total RNAs (200ng) were then reverse transcribed into complementary DNA (cDNA) using High-Capacity cDNA Reverse Transcription Kit (Applied Biosystems, Waltham, Massachusetts, USA) following the manufacturer’s instructions. Transcription products (10ng) were amplified by qPCR using SYBR Takyon Mix (Eurogentec, Seraing, Belgium) on StepOne Plus™ system (Applied Biosystems, Waltham, Massachusetts, USA) using primers detailed in Table 3. Data analyses were performed with the StepOne™ software v2.3 (Applied Biosystems, Waltham, Massachusetts, USA). Target transcript levels (N-target) were normalized to the housekeeping gene (*16SRNA*) transcript levels and mRNA level with the equation N-target = 2^∆Ct, where ∆Ct is the Ct value of the target gene after subtraction of Ct for the *16SRNA* gene. At least three independent experiments were performed for each *C. acnes* strain.

**Table 3:** Nucleotide sequences of *C. acnes* genes primers used for qPCR

	Gene	Function	Upper	Lower
*Housekeeping*	*16SRNA*		TTATTGGGCGTAAAGGGCTC	CCCCTACCTTCCTCAAGTCA
	*lexA*	*SOS response*	CCAAGGAACGCGAAGTCTT	GCTGGACTGTTGATGTGCT
*Stress-induced*	*recA*	*SOS response*	TCACCAGCAGGGAATCTGTA	TCACCAGCAGGGAATCTGTA
	*PPA0387*		AAGCTCCGCGAAACCATTAT	CAACCTGATCAGACTGGTCG
	*PPA2127*	*Adhesin*	CACCTCAAGAAAGCAGCCA	CCAAGTGGGTGACGATACG
	*CAMPI*	*Adhesin*	TTCTACACCAAGACCAAGCTG	GCACGGCTCATGAAGTAAATG
	*dnaK*	*Chaperone protein*	GGAGGTGTTCACTACTGCTG	GGCATAAGACCAGTCAGCTC
*Biofilm*	*groEL*	*Chaperone protein*	CGTTACCAAGGACAACACCA	AGTCCGAGTTGTCGTACTCA
*initiation and formation*	*fusA*	*Proteins biosynthesis*	TGAACTCGGTGCTCATTACG	CTCGAGGTAGAGCTCCATGA
	*tuf*	*Proteins biosynthesis*	ATCTCGACAAGCCCTTCCT	TACCGACGATCTCGACCTC
	*eno*	*Glycolysis*	CGAAGAAGCTTGGTGAGAAGA	AATCTGGTTCACCTTGACGAG
	*fumC*	*TCA cycle*	CCAAACCCCGACAAAATCAAG	TTCTTAGCGATCTTCGAAGCC
	*SucD*	*TCA cycle*	ATCATTAGCCCCGGGAAGT	ACCTCGTACATGAGCTGGT

### *C. acnes* internalization by SaOS-2

Osteoblast cell line SaOS-2 (ATCC HTB-85™) used for this study, were cultivated in medium composed of Dulbecco’s Modified Eagle Medium (DMEM, Thermo Fisher Scientific, Waltham, Massachusetts, USA) supplemented with 10% fetal bovine serum (FBS, PAN-Biotech GmbH, Aidenbach, Germany) and 1% Penicillin Streptomycin (PS, Thermo Fisher Scientific, Waltham, Massachusetts, USA). The bacterial internalization experiment was adapted from Josse J. et al., 2016 [24]. Briefly, SaOS-2 were seeded in 24-well plates at 10^4^ cells/cm² and incubated at 37 °C, in a 5% CO_2_ humidified atmosphere, for 72 h, in culture medium. SaOS-2 cells were washed twice with PBS and incubated overnight with PS-free culture medium. The following day, cells were again washed twice with PBS and 1 mL of PS-free culture medium was added in each well. One well was used for evaluation of the number of cells per well. Cells were detached using trypsin-EDTA (Thermo Fisher Scientific, Waltham, Massachusetts, USA) and 10 µL were deposited on Kova slide for counting. Bacteria were centrifugated for 5 min, at 5000 g and the pellets were rinsed twice with PBS. Bacteria were then added to cell cultures to obtain a multiplicity of infection (MOI) of 100:1. After 3 h of interaction, cells were washed twice with PBS and incubated with cell medium containing 100 µg/mL of gentamicin (Fisher Scientific, Hampton, New Hampshire, USA), during 1 h, at 37 °C, in a 5% CO_2_ humidified atmosphere, to eliminate extracellular bacteria. Cells were again washed twice with PBS, and 1 mL of 0.1% Triton X-100 solution was added in each well, during 5 min, to harvest intracellular bacteria. Lysate were seeded on blood agar plates using automatic seeder EasySpiral (Interscience, Saint-Nom-la-Bretèche, France) and incubated at 37 °C under anaerobic condition for 5 days. The number of recovered CFU was determined using automatic counter SCAN 1200 (Interscience, Saint-Nom-la-Bretèche, France) and the percentage of internalized-bacteria was defined as follows: % of bacteria = ((CFU/mL)/(number of cells x MOI)) x 100. Four independent experiments were performed.

### Graphical representation and statistical analysis

Results are represented as whisker plots: the box represents the interquatile range and the whiskers are the minimum and the maximum values, the bar inside the box is for the median value, and each point represents an independent replicate. The average value is indicated by a red bar (or a black bar for Live/Dead graphes). All up- and down-fold variations between conditions were expressed from average values. The statistical significance of the results was assessed using the exact 2-tailed non-parametric Wilcoxon-Mann-Whitney test of GraphPad Prism (v8.0.1) software. Non-parametric statistics were used owing to the lack of a normal distribution of the assessed variables. Differences were considered significant at *p* < 0.05.

## Results

### Titanium supports favor *C. acnes* adhesion

**Fig. 1:**
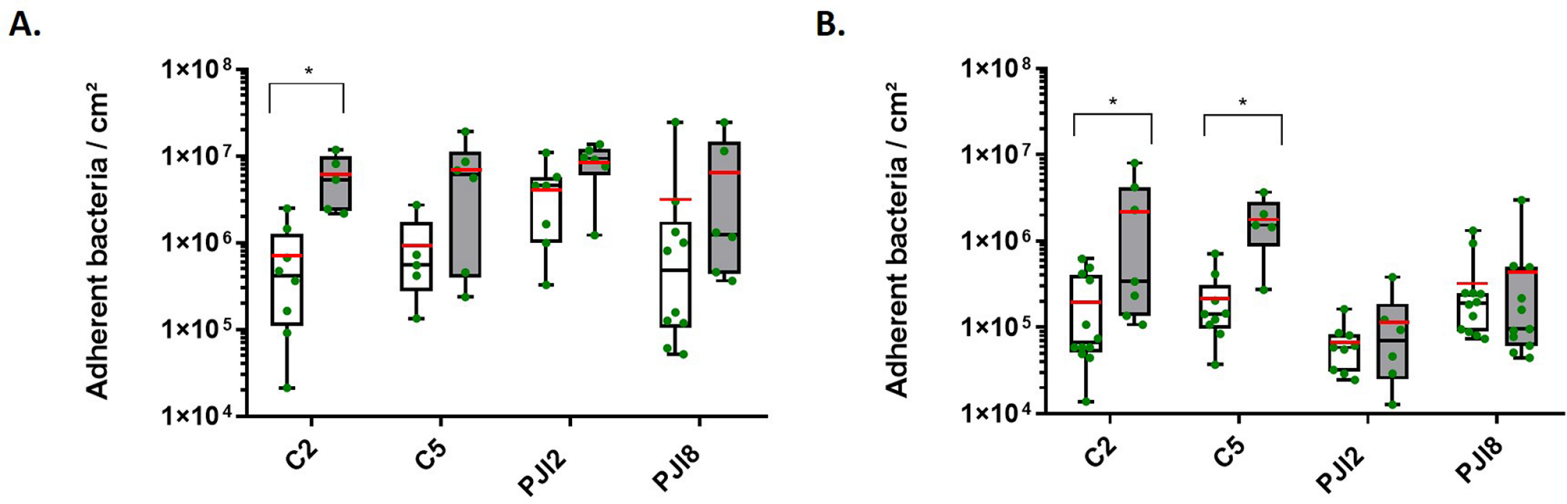
*C. acnes* adherence increases on textured titanium disk with nutritive or diluted broth **(A)** Quantity of adherent bacteria cultivated for 5 days with BHI or **(B)** with dBHI. White bars represent the number of bacteria adhered on plastic coverslips, grey bars represent the number of bacteria adhered on textured titanium disk and green points represent each independent biological replicate. The average value is indicated by a red bar. Wilcoxon-Mann-Whitney test: * *p* < 0.05. Experiment was performed at least 3 independent times. Name of strains: C is for non-PJI-related strain and PJI is for PJI-related strain

In BHI medium, C2 strain adhered 8.4-fold more on textured titanium disk as compared to plastic coverslip by up (Fig. 1A). No impact of the support’s nature was observed on planktonic growth (see Additional file 1A). As expected, in diluted BHI medium (dBHI), there was a weaker planktonic growth (see Additional file 1B) and a lower number of adhered bacteria. Nevertheless, non-PJI-related strains, C2 and C5, adhered significantly more on textured titanium disks than on plastic coverslip (11.2 and 8.2-fold increase respectively) (Fig. 1B).

As the surface texturing of titanium is an additional difference between plastic and titanium, bacterial adhesion was also evaluated on raw titanium (no texturing) in both condition (BHI and dBHI) (see Additional file 2). No significant differences in adhesion were observed between raw and textured surfaces (Fig. 1A and B and see Additional file 2).

### Titanium disks triggers aggregates biofilm formation with polysaccharides and proteins enriched matrix

**Fig. 2:**
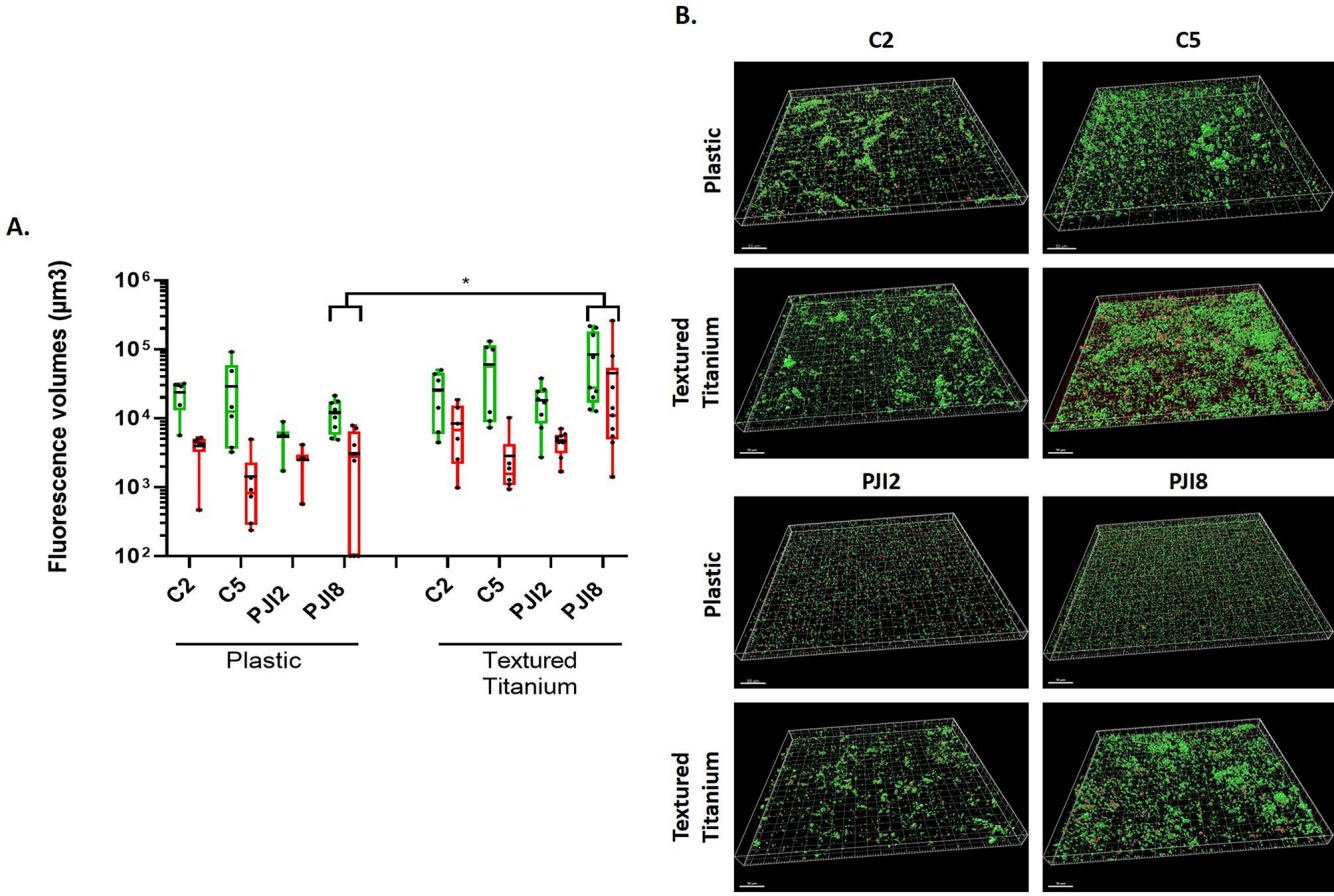
Textured titanium changes proportion of live and damaged bacteria and biofilm morphology. **(A)** Fluorescence volumes of live and adherent bacteria, labelled by SYTO9™ (green) and damaged and dead bacteria, labelled by PI (red) according to the support. The average value is indicated by a black bar. **(B)** 3D-representative reconstruction of *C. acnes* biofilm. Wilcoxon-Mann-Whitney test: * *p* < 0.05. Experiment was performed 3 independent times

To go further, *C. acnes* biofilm in dBHI was observed thanks to CLSM. The quantity of bacteria was more important on textured titanium disk than on plastic coverslip whatever the origin of strains. The difference was more obvious for PJI8 (Fig. 2A). Moreover, we noticed a similar difference between raw titanium and plastic coverslip for PJI-related strains (see Additional file 3 A).

Thanks to 3D-reconstruction, large bacteria aggregates for C2 and C5, on all materials and small aggregates for PJI8 on raw titanium were observed (Fig. 2B and see Additional file 3B). For PJI-related strains, aggregates were mainly present on textured titanium disk (Fig. 2B).

**Fig. 3:**
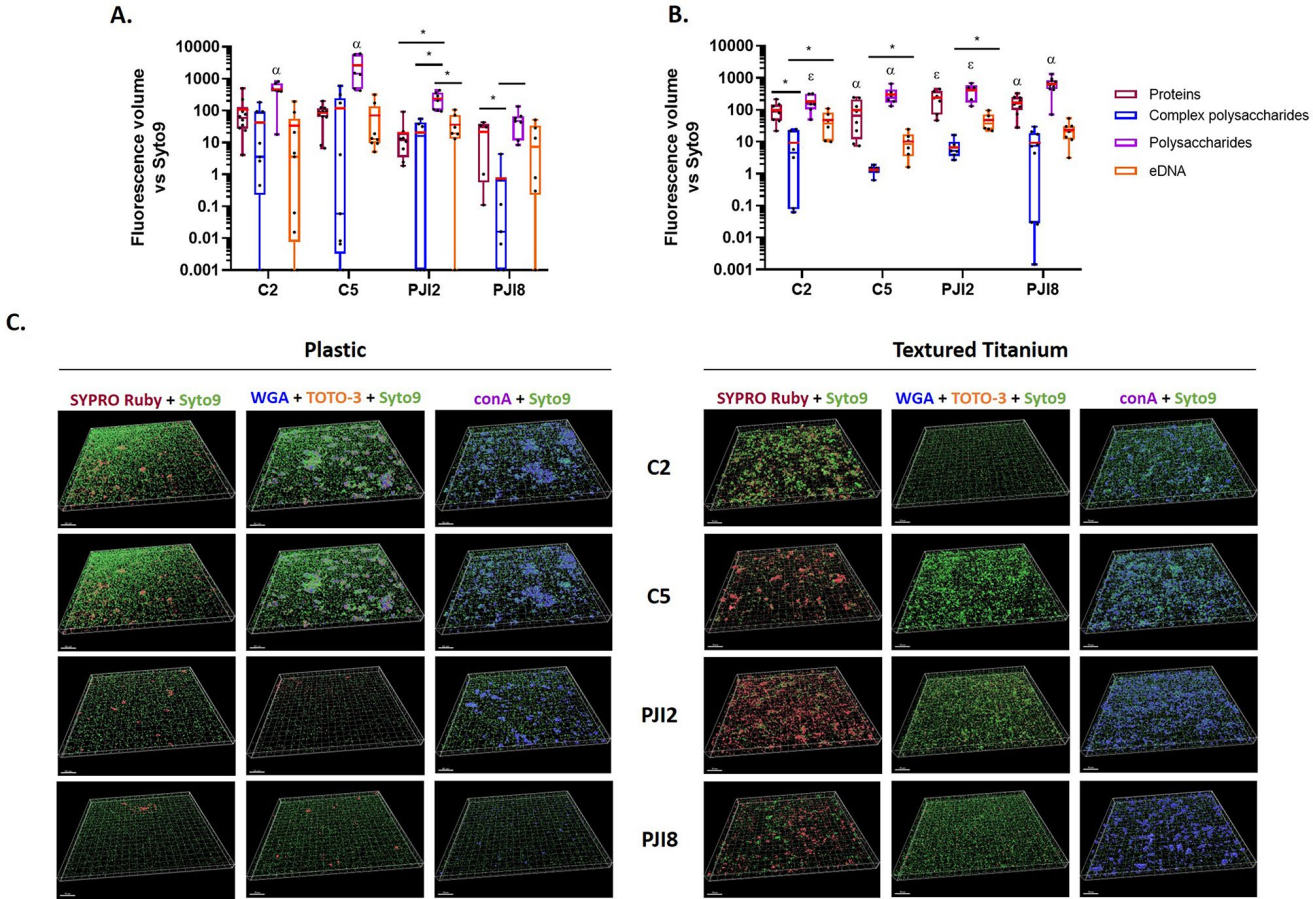
*C. acnes* biofilm composition on textured titanium disk is mostly composed by polysaccharides and proteins. **(A)** *C. acnes* biofilm composition on plastic coverslip and **(B)** on textured titanium disk after 5 days with dBHI. The fluorescent volumes of the matrix components: SYPRO Ruby^®^ for proteins (dark red), Wheat Germ Agglutinin (WGA) (blue) and concanavalin A (conA) (purple) for complex and simple polysaccharides respectively, and TOTO-3™ for extracellular DNA (orange) were normalized to fluorescent volumes of SYTO9™ using IMARIS software after acquisitions by CLSM. The average value is indicated by a red bar. Wilcoxon-Mann-Whitney test: *p* < 0.05, * between fluorescent labels, α *versus* each fluorochromes and ε *versus* WGA/TOTO™. **(C)** 3D-views of matrix biofilm composition of *C. acnes*. Experiment was performed 3 independent times

On plastic coverslip, the extracellular components were mainly simple polysaccharides, in particular for non-PJI-related strains. PJI8, which produced lower relative amount of complex polysaccharides than other strains. The other components were in similar lower proportions (Fig. 3A and C). On textured titanium disk, the relative amount of fluorochrome signals were similar between proteins and simple polysaccharides, and lower for eDNA and complex polysaccharides (Fig. 3B and C). On raw titanium disk, these same relative amounts were also observed in biofilm formed, except for PJI2 biofilm which showed a more important part of eDNA compared to textured titanium disk (see Additional files 3 C and 3D).

### Stress and biofilm-formation-related genes expression by *C. acnes* is reduced on titanium support

According to previous publications [20, 21], a list of genes involved in *C. acnes* biofilm initiation and formation was defined. We compared the expression of these genes on plastic coverslip and on titanium disks.

**Fig. 4:**
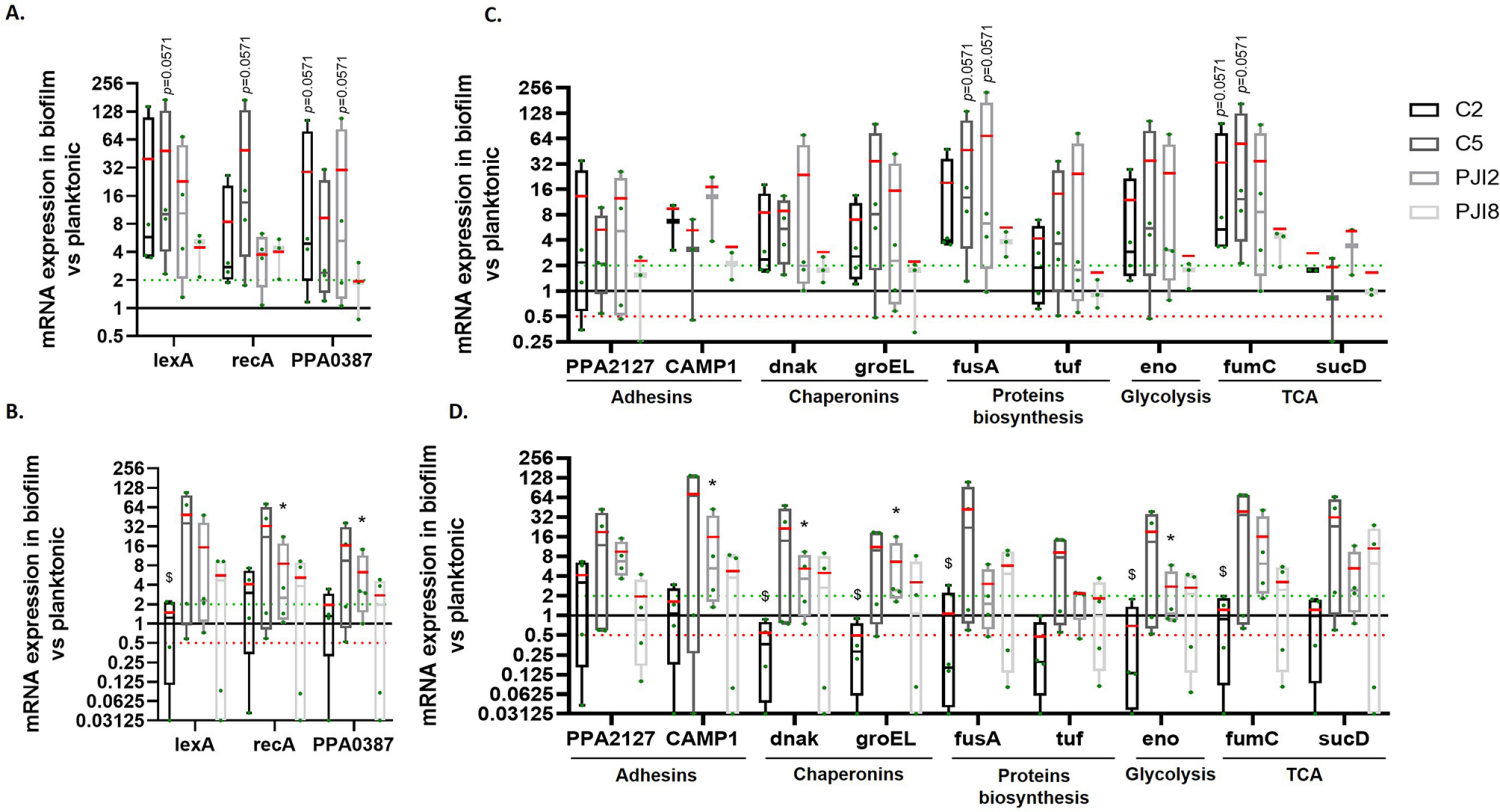
mRNA expression of genes is modified for one non-PJI-related strain on textured titanium disk. **(A)** Difference of expression of stress pathway-related genes when biofilm is formed on plastic coverslip and **(B)** on textured titanium disk. **(C)** Levels of expression of gene involved in biofilm formation on plastic coverslip and **(D)** on textured titanium disk. The average value is indicated by a red bar. Experiment was performed at least 3 independent times. Wilcoxon-Mann-Whitney * *p* < 0.05 expression in biofilm *versus* expression in planktonic counterpart, $ *p* < 0.05 textured titanium *versus* plastic support

First, genes involved in stress response and biofilm ignition were studied. On plastic coverslip, an activation of genes was observed for non-related-PJI strains and PJI2 with an increase of *PPA0387* expression in biofilm compared to planktonic for C2 and PJI2 and an increase of SOS response genes expression for C5 (Fig. 4A). Conversely, no variation of *recA*, *lexA* and *PPA0387* was quantified for PJI8 in bacteria within biofilm compared to planktonic counterparts (Fig. 4A). On textured titanium disk, the profile of gene expression was similar except for C2 strain with notably a slight or an absence of activation (Fig. 4B). The profile of gene expression was the same on textured and raw titanium disks for PJI-related strains, but different for non-related PJI strains with a lower expression of the genes without being significant (see Additional file 4A).

Second, the genes involved in biofilm formation were also evaluated [20, 21]. On plastic coverslip, *CAMP1*,* fusA*, and *fumC* are up-regulated in the biofilm of the four *C. acnes* strains (with an increase of *fumC* expression for non-related-PJI strains in biofilm compared to planktonic and of *fusA* for C5 and PJI2) (Fig. 4C). However, a biofilm-related overexpression of *dnaK*, *eno*, *PPA2127* and *groEL* was observed in C2-, C5- and PJI2- biofilm, on plastic coverslip (Fig. 4C). In comparison, on textured titanium disk, gene response was more variable. For the non-PJI-related strain C2, no change of gene expression involved in biofilm was identified except the observation of a down-regulation of gene *PPA2127* coding for an adhesin (Fig. 4D). PJI-related strains and C5 tended to exhibit increased expression of most of biofilm related genes notably for *CAMP1*, *dnak*, *groEL* and *fumC* for PJI2 with an expression significantly more important in biofilm compared to planktonic (Fig. 4D). A similar profile of gene expression was observed on raw titanium disk except for the non-PJI-related strain C5 with an expression lower on raw titanium than on textured titanium (see Additional file 4B).

Globally, gene expression level for C2 are statistically lower on textured titanium than on plastic for *lexA*,* dnak*, *groEL*, *fusA*, *eno* and *fumC*.

To summarize, *C. acnes* behavior changed according to the support with: i/ a greater adhesion, and ii/ a biofilm composed by both proteins and polysaccharides and iii/ a strain dependent gene response on titanium disk compared to plastic coverslip. As no notable difference was observed between raw and textured titanium, only textured titanium was used on the further experiments.

### *C. acnes* PJI-related strains adhesion is better on titanium after osteoblast internalization

**Fig. 5:**
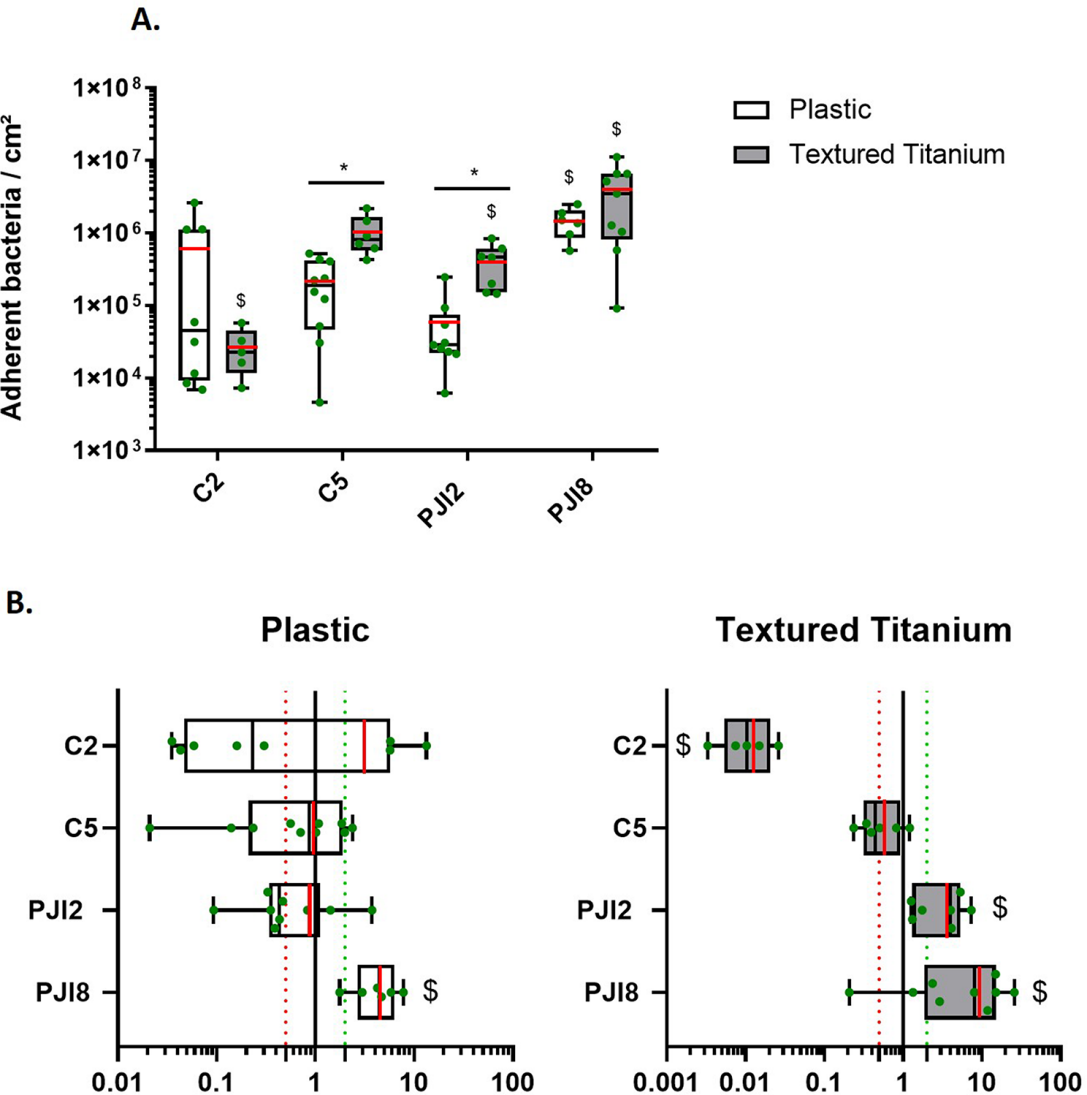
Internalization by SaOS-2 increases PJI-related strains adhesion on textured titanium disk. **(A)** Quantity of adherent bacteria, after internalization, cultivated with dBHI. **(B)** Ratio after/before internalization. White bars represent the number of bacteria adhered on plastic coverslip, grey bars represent the number of bacteria adhered on textured titanium disk and green points represent each independent biological replicate. The average value is indicated by a red bar. Wilcoxon-Mann-Whitney test: *p* < 0.05, * between supports and $ *versus* non-internalized *C. acnes*. Experiment was performed after 4 independent internalization

The internalization has especially impacted C2 and PJI8 bacterial adhesion (Fig. 5A). Adverse response was observed for C2 strain between plastic and textured titanium with a significant decrease (80.2-fold down) on titanium and a not significant increase on plastic compared to non-internalized strain (Fig. 5B). A significant increase was observed for PJI8 on plastic coverslip (4.5-fold up) and on textured titanium (9.2-fold up for PJI8) (Fig. 5B).

### Internalization of *C. acnes* into osteoblasts affects biofilm composition according to the strain and the support

The composition of biofilm formed by bacteria that had been internalized by SaOS-2 was analyzed.

**Fig. 6:**
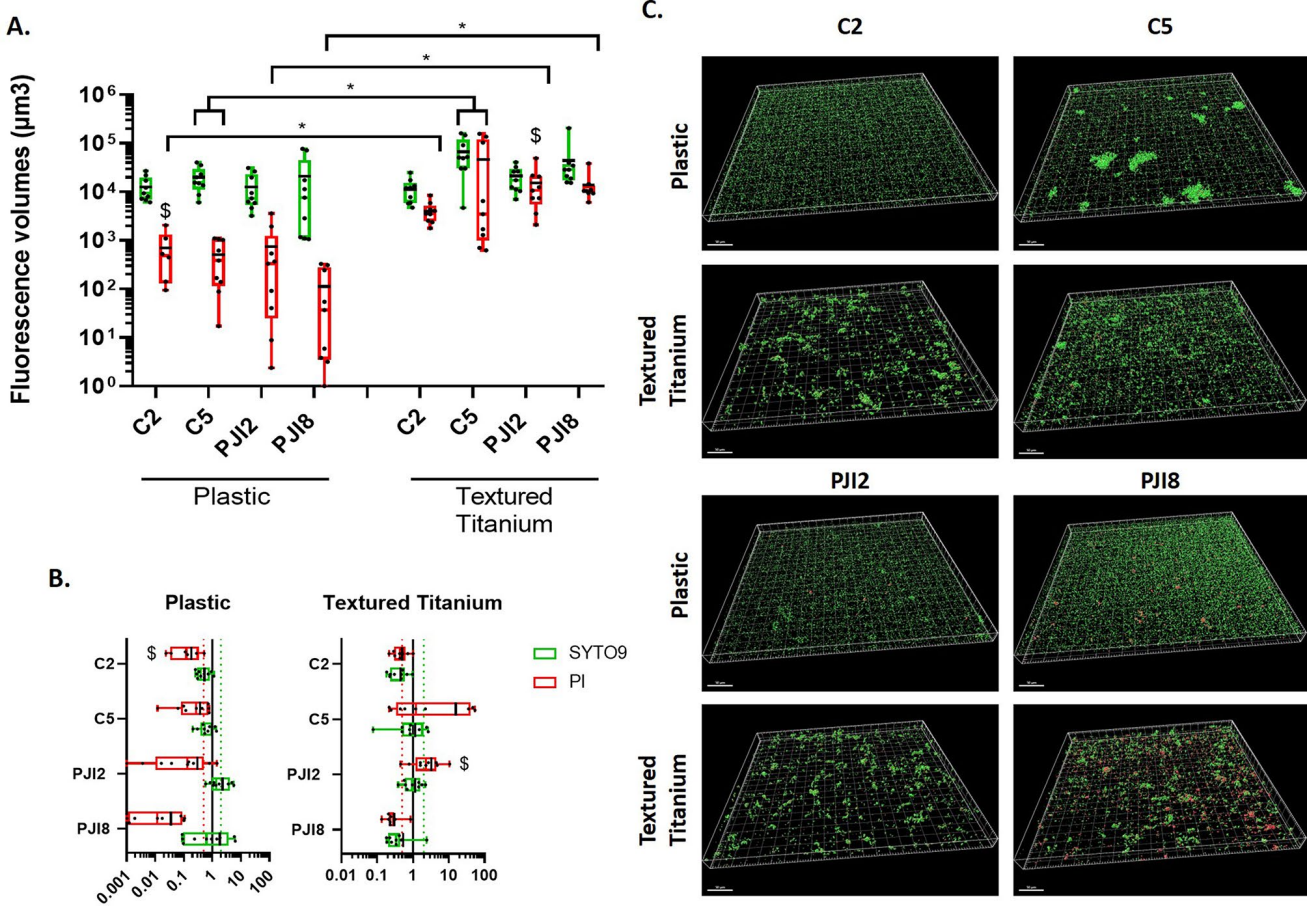
Internalization slightly modified the proportion of damaged and dead bacteria and biofilm morphology. **(A)** Fluorescence volumes of live and adherent bacteria, labelled by SYTO9™ (green) and damaged and dead bacteria, labelled by PI (red) according to the support. **(B)** Ratio after/before internalization of fluorescence volumes. The average value is indicated by a black bar. **(C)** 3D-representative reconstruction of *C. acnes* biofilm. Wilcoxon-Mann-Whitney test: *p* < 0.05, * plastic *versus* textured titanium and $ *versus* non-internalized *C. acnes*. Experiment was performed 3 independent times

Compared to non-internalized *C. acnes* strains, a significant decrease of damaged and dead bacteria was observed for internalized-C2 and a non-significant decrease for the other strains on plastic coverslip (Fig. 6A and B). On textured titanium disk, damaged and dead bacteria fluorescent labeling was more important for internalized-C5 and -PJI2 (3.3 to 15.8-fold up) with a significant difference for PJI2, compared to non-internalized strains (Fig. 6B).

Thanks to 3D-reconstruction, a loss of bacteria aggregates was only observed for internalized-C2 compared to non-internalized-C2 (Fig. 6C). Biofilm morphology on textured titanium disks was not modified by internalization for any strains (Fig. 6C).

**Fig. 7:**
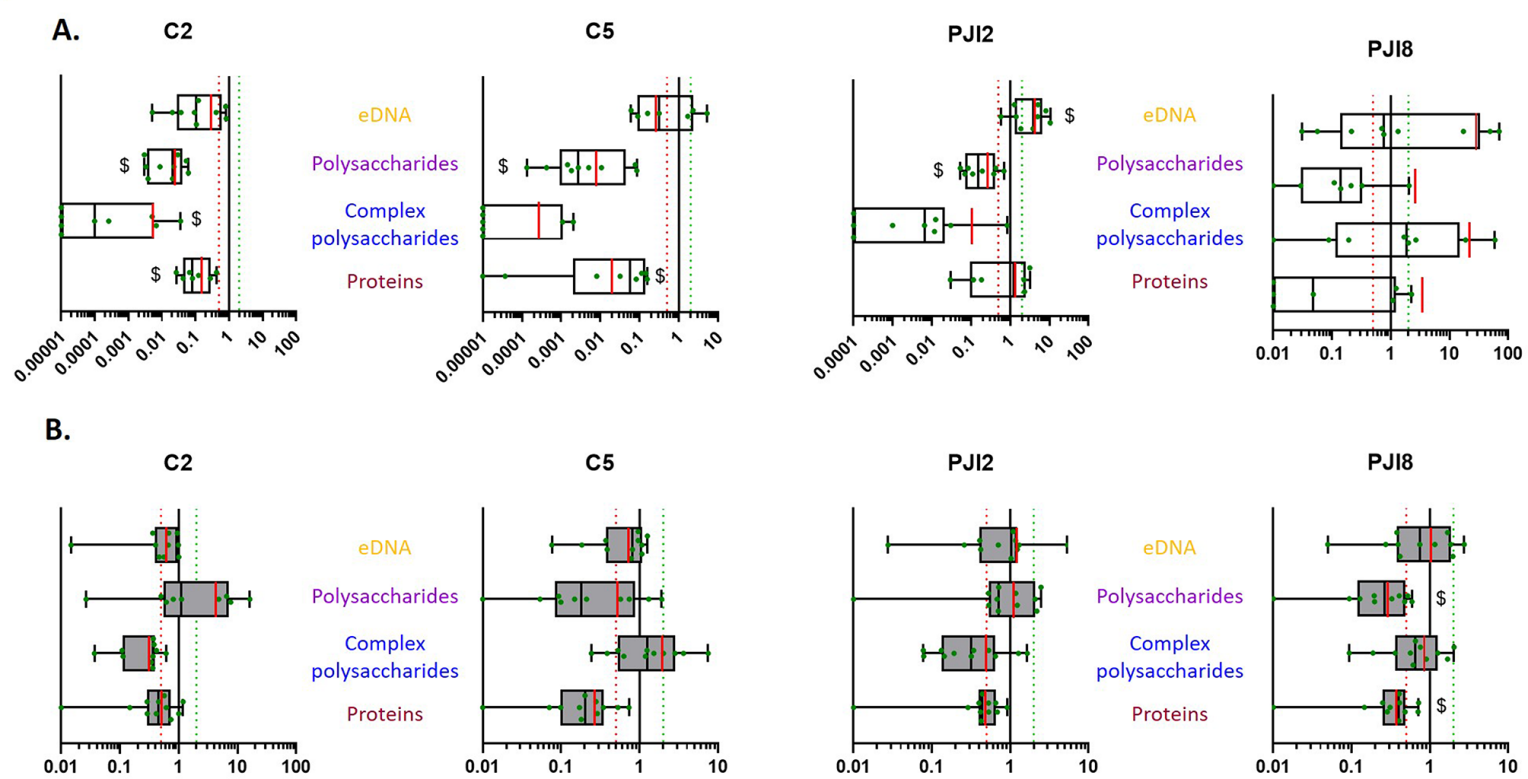
Internalization induces modifications on matrix biofilm composition. **(A)** Ratio after/before internalization on plastic coverslip and **(B)** textured titanium disk. Fluorescence volumes matrix component were normalized by SYTO9™. The average value is indicated by a red bar. Wilcoxon-Mann-Whitney test realized on all data *versus* non-internalized *C. acnes* $ *p* < 0.05. Experiment was performed 3 independent times

On coverslip, non-PJI-related strains formed biofilms with lower levels of proteins (14.9 to 16.4-fold down) and polysaccharides (10.7 to 52.2-fold down) after internalization (Fig. 7A). For PJI-related strains, the biofilm was mostly composed of eDNA (4.2 to 15.5-fold up) with a significant increase for PJI2 on plastic coverslip (Fig. 7A). On textured titanium disk, a decrease of fluorescent signal of proteins was observed for all internalized-strains (1.8 to 3.5-fold down). An increase of labeled-polysaccharides was quantified for C2 (2.9-fold up). Conversely, a significant decrease of polysaccharides was noted for PJI8 after internalization compared to non-internalized strains (3.1-fold down) (Fig. 7B).

Briefly, after internalization, on plastic, biofilm is composed by the same relative amount of all components except for complex polysaccharides for non-PJI-related strains and mostly by eDNA for PJI-related strains (see Additional files 5 A and 5 C). On textured titanium disk, polysaccharides were the main components observed in the biofilm of all internalized-*C. acnes* strains (see Additional files 5B and 5 C).

### Internalization of *C. acnes* into osteoblasts blunts mRNA expression for stress and biofilm formation-related genes except for C2 strain on titanium support

**Fig. 8:**
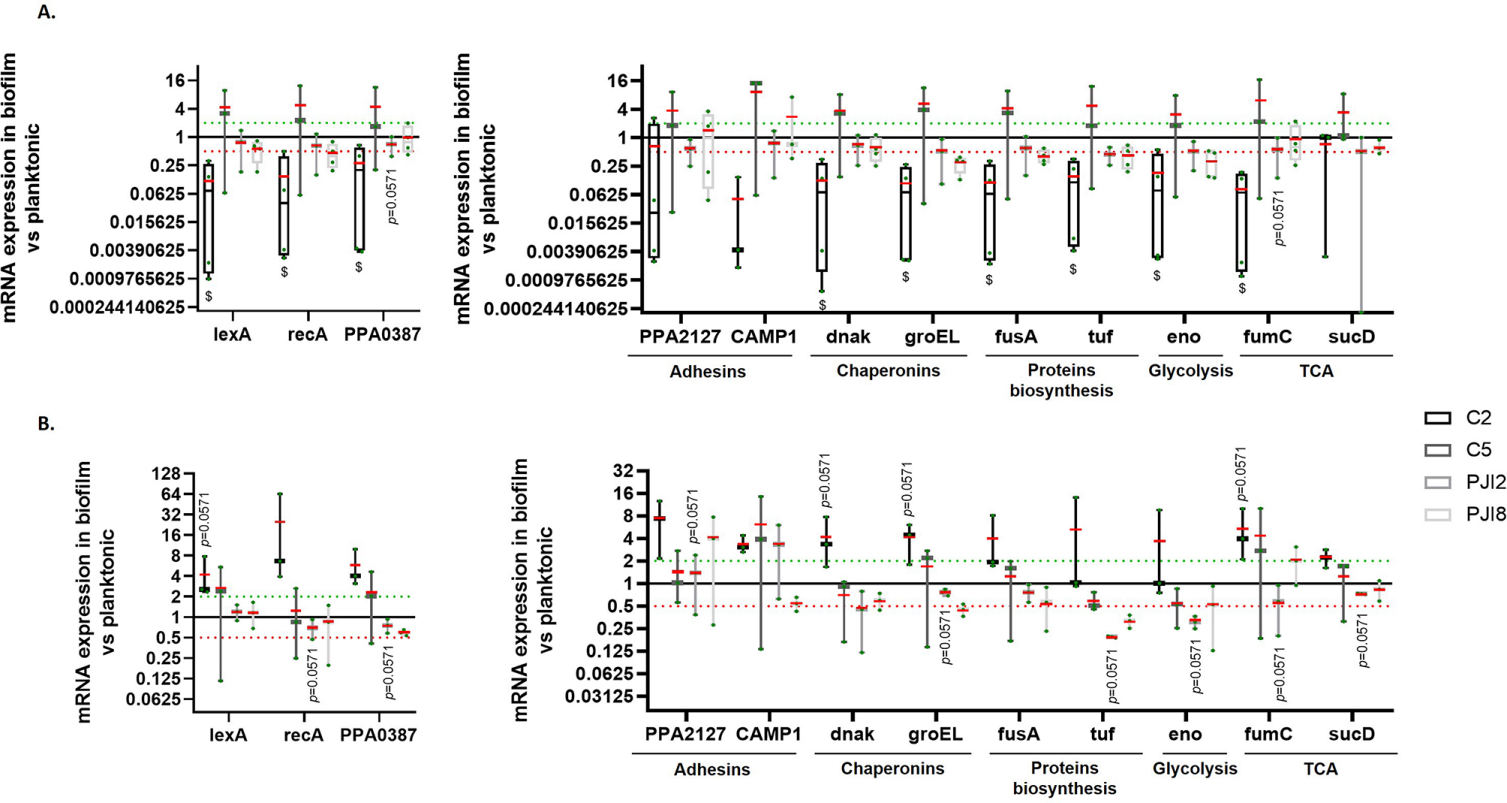
mRNA expression of genes is activated in C2 biofilm on textured titanium disk after internalization. **(A)** Levels of expression of stress-induced genes and genes involved in biofilm formation on plastic coverslip and **(B)** textured titanium disk. The average value is indicated by a red bar. Wilcoxon-Mann-Whitney test, $ *p* < 0.05 compared to non-internalized strain. Experiment was performed at least 3 independent times

The levels of genes expression in biofilm *versus* planktonic bacteria, after internalization, were compared to non-internalized bacteria. On plastic coverslip, a decrease of all biofilm induction- and formation-genes was observed for C2 (significantly different for the majority of genes) and PJI2 (Fig. 8A). On textured titanium disk, a decrease of stress-induced genes and biofilm-induced genes was observed for PJI-related strains (notably for the majority of genes for PJI2) and the non-PJI-related strain, C5 (Fig. 8B). For internalized-C2, an important increase of all genes was noted on textured titanium disk (notably for chaperonins *dnak* and *groEL* and for *fumC*) (Fig. 8B). 

## Discussion

While PJI remain a major clinical problem, the infectious mechanisms of *C. acnes* are still unclear [25] and no host inflammation markers is usually detected [26, 27]. The biofilm formation could be the key to this clinical situation, protecting bacteria from immune system attack and antimicrobial treatment. However, the biofilm structure and composition are strongly environment- and support-dependent [16, 22]. The study of *C. acnes* biofilm on plastic support could thus not be adapted or even inaccurate. The aim of this study was to characterize *C. acnes* biofilms in PJI context, by comparing non-PJI-related and PJI-related strains behaviors on plastic (classic in vitro model) and titanium surfaces (prosthesis-mimicking material), and by analyzing the impact of bacterial internalization in host cells on further biofilm formation.

Bacterial adhesion is the first step of biomaterial-related infections, and it paves the way to implant colonization [28]. The adhesion of *C. acnes* on different metal alloys was previously studied and demonstrated metal-dependent biofilm structures [15]. In PJI context, *C. acnes* colonize the bone and prosthesis interface where the nutrient availability is poor (cortical bone, characterized by low-vascularized tissue), therefore biofilm being strongly dependent to its environment [22], a more faithful model to the PJI context using titanium surfaces and poor media was developed.

In our model, differences in bacterial adhesion were observed between the plastic coverslip and titanium disk: non-PJI-related strains adhered more to textured (or raw) titanium than to plastic coverslip. Although the difference was not significant for PJI-related strains after enumeration of adherent bacteria on agar plates, a significant difference in SYTO-9 and IP-labelled adherent bacteria quantity was observed on raw and textured titanium compared to plastic for PJI8. Variations between those two technics for live and adherent bacteria can be explained by the presence of bacteria under different metabolic states in biofilm, like persister, viable bacteria non-cultivable (VBNC), or dormant bacteria are labeled by fluorochromes but are not detectable by classical culture assay. The live and dead staining observation revealed another difference between non-PJI- and PJI-related strains as the second ones did not form aggregates on plastic. Overall, this reinforces the hypothesis that titanium and plastic surfaces influence *C. acnes* biofilm formation differently. Titanium surface texturing does not appear to affect bacterial adhesion as no difference was observed between raw and textured titanium. The IP labeling, notably more important for PJI8 on titanium compared to plastic, permitted to observe an importance of bacterial lysis. This phenomenon leads to the release of eDNA essential in biofilm matrix formation.

Indeed, another important characteristic of biofilm is the composition of the matrix, made of extracellular components. In our study, we observed that biofilm matrix is mostly composed of polysaccharides (2.4 to 30-fold up compared to proteins) in the biofilm of all *C. acnes* strains on plastic coverslip, with a slightly weaker signal of eDNA compared to proteins. These data confirmed previous studies analyzing biofilms formed on plastic [20, 29]. Gannessen AV. et al., 2019, showed a composition of 62.6% of sugars, 9.6% of proteins, and 4% of eDNA in RT5 (acneic strain) biofilm on Petri dishes by dosing compounds [19]. The presence of an almost equivalent signal of proteins and eDNA can be explained by the importance of these compounds on *C. acnes* adhesion. Indeed, Kuehnast T. et al., 2018, showed that eDNA and proteins play a role in *C. acnes* adhesion and biofilm maturation on plastic by using proteinase-K and DNAse-I enzymes treatment for several *C. acnes* strains with different phylotypes [17]. Here, on titanium disks, the proportions of polysaccharides and proteins in *C. acnes* biofilm were more balanced (0.3 to 1.7-fold up on raw titanium disk and 1.7 to 3.8-fold up for polysaccharides compared to proteins). The presence of a strong protein signal compared to eDNA could also lead us to hypothesize that proteins are more important than eDNA for the biofilm matrix production of *C. acnes* on titanium. Proteomic studies will have to be carried out to identify which proteins are mainly involved in matrix composition.

*C. acnes* biofilm being studied only recently, few proteomic and transcriptomic experiments have been carried out [20, 21]. Based on literature, selected genes identified as important for biofilm formation were monitored for their expression in biofilm. We confirmed that these genes are globally induced in biofilm formed on plastic, for all the four strains. Interestingly, the involvement of these genes is different in the biofilm formed on titanium, especially for non-PJI-related strains, showing that other genes, not yet identified, play a role for adhesion on titanium.

SOS response genes (*recA* and *lexA*) were strongly expressed in biofilm formed on plastic compared to planktonic bacteria, contrary to their expression in biofilm formed on titanium, which was strain-dependent. The genes involved in proteins biosynthesis (*fusA* and chaperone *dnak*), glycolysis (*eno*), TCA cycle (*fumC*) and *CAMP1* (recognized by TLR-2, an eucaryotic cell membrane receptor) were overexpressed for all strains in the biofilm formed on plastic. These results correlated with the strong presence of proteins in the biofilm matrix composition. On titanium disks, the overexpression of the two genes coding for chaperones proteins (*dnak* and *groEL*) and *fusA* for PJI-related strains showed the importance of proteins for biofilm on titanium. Other genes involved in the synthesis of matrix components were also overexpressed (*sucD*, *eno*, *fumC*) for PJI-related strains. However, the absence of overexpression of these genes in the non-PJI-related strain C2 with a strong down-regulation even being observed underlined a strain-dependent response which could be related clinical context. Thus, we speculate the presence of different proteins with a role in the matrix composition or adhesion between PJI-related and non-PJI-related strains or according to the phylotype, and differently produced according to the nature of the support.

In PJI context, bacteria can interact with host cells. Aubin A. et al., 2017, has shown the capacity of MG63 osteoblast cells to internalize *C. acnes* [8]. Mongaret C. et al., 2020, confirmed this result with SaOS-2 cell line, showing that internalization increased biofilm formation of some *C. acnes* strains after intracellular extraction [9]. Dubus M. et al., 2020, had also observed that *C. acnes* internalization in bone marrow derived mesenchymal stem cells affects their behavior, leading to an increase of biofilm formation on orthopedic material (Titanium alloy and PEEK) when bacteria are released out of the cells [10].

We observed the same phenomenon in our model for PJI2 on titanium and PJI8 on both supports. On contrary, we observed that *C. acnes* internalization in SaOS-2 influenced adhesion of C2 strain, with a decrease of adhered cells on titanium alloys and a non-significant increase of polysaccharides quantity within the matrix. This increase can be also correlated to an overexpression of *eno*, involved in glycolysis. Some of the genes involved in protein biosynthesis were strongly expressed in biofilm formed in titanium by internalized C2 compared to the non-internalized one. It could reflect the production of adhesins that could play a different role in bacterial adhesion on titanium. The behavior of internalized C2 strain was also modified on plastic but in a different way: no more aggregates were observed and the quantity of dead cells within biofilm decreased as well as the overall quantity of extracellular components. These modifications could be linked to a strong decrease of the expression of all tested genes involved in biofilm formation on plastic compared to non-internalized C2 bacteria. We conclude that internalization could strongly affected the behavior of specific *C. acnes* strains like observed for C2 strain. However, the difference between both supports reinforce the hypothesis that *C. acnes* metabolic pathways involved toward titanium or plastic support are different.

For the three other strains, the internalization affected the quantity of extracellular components by increasing or decreasing the different components levels in a strain-dependent way and especially for biofilms formed on plastic. Thus, on plastic, a decrease of polysaccharides in biofilm was observed for all internalized strains (except complex polysaccharides for PJI8) following internalization, with a rise of eDNA for PJI-related strains. These modifications were not found in biofilm formed on titanium after internalization. The internalization influenced the matrix biofilm by increasing the relative amount of polysaccharides on textured titanium disk. Expressions of genes known to be involved in biofilm formation was modified similarly for the two PJI-related strains and the non-PJI-related strain C5 but differently for C2 strain. These results suggest that different strains responses to bacteria internalization by osteoblasts affect the gene expressions and the future matrix composition involved in biofilm formation. Different hypotheses could be raised as a modification of cell wall [30]. The modification of bacterial cell wall could affect adhesins composition and interactions with support. We speculate that post-translational modifications could also be induced within the host cell modifying or activating enzymes involved in different pathways such as proliferation or synthesis of matrix components. Moreover, as the C2 strain does not respond the same as the C5 strain, the hypothesis of the existence of a link between phylotype and internalization can be asked.

We confirmed that the impact of internalization is strain-dependent. The non-PJI-related strain, C2, does not react in the same way to internalization that the other strains. Dubus M. et al., 2022, have shown modifications of elasticity of bacteria cell wall and of IR spectrum of non-PJI-related *C. acnes*, which can suggest a probable decrease of polysaccharides after internalization by MSCs, as we observed in this study [30]. Therefore, we speculate that a structural modification of *C. acnes* surface or an adaptive response during internalization could lead to the establishment of a modification in biofilm organization of non-PJI-related *C. acnes*.

The differences observed between titanium and plastic showed that the bacteria do not trigger an universal response but specific ones, regarding the support. This study therefore shows the importance of the model to choose to study the biofilm of *C. acnes*, such as the use of prosthesis-mimicking titanium in the investigations of PJIs. The interaction of bacteria with bone cells (osteoblasts) induces modification of *C. acnes* behavior demonstrating the importance of bone environment parameters to be included in in vitro model of PJI. Previous studies have also shown that *C. acnes* is able to adhere to fibronectin, one of the main components of the bone matrix [31]. However, it is also necessary to consider the presence of the bone cement used during the bone-prosthesis surgeries, which can prone *C. acnes* adhesion [7] as well as the type and quality of bone another important element to develop an accurate model. In a recent study, Lamret F. et al., 2023, has shown that *S. aureus* is able to adhere and form a biofilm on decellularized bone explant [32]. Thus, the influence of other supports as bone explant and bone cement have to be explored in future.

## Conclusion

In conclusion, our results showed that *C. acnes* adapts its biofilm formation and composition according to the microenvironment. In a strain dependent way, *C. acnes* were strongly influenced by the nature of the physical materials mimicking bone prosthesis but also by internalization in osteoblasts, both representing the bone-prosthesis environment. These differential and complex responses emphasize the need to develop a relevant in vitro model toward bone-prosthesis site. The description of the biofilm characteristics formed on titanium, or after contact with osteoblasts, will help to choose the right treatment to fight against this form of bacterial persistence.

## Electronic supplementary material

Below is the link to the electronic supplementary material.

Supplementary Material 1

Supplementary Material 2

Supplementary Material 3

Supplementary Material 4

Supplementary Material 5

Supplementary Material 6

## Data Availability

The data generated and analyzed during the study are available from the corresponding author upon reasonable request.

## Abbreviations

BHI: Brain Heart Infusion
*C. acnes*: *Cutibacterium acnes*
CFU: Colony Forming Unit
ConA: Concanavalin A
CSLM: Confocal Scanning Laser Microscopy
DMEM: Dulbecco’s Modified Eagle Medium
FBS: Fetal Bovine Serum
MOI: Multiplicity of infection
MSC: Mesenchymal stem cell
PBS: Phosphate Buffered Saline
PCR: Polymerase Chain Reaction
PEEK: Polyether ether ketone
PI: Propidium Iodide
PJI: Prosthesis Joint Infection
PS: Penicillin Streptomycin
TCA: Tricarboxylic Acid Cycle
VBNC: Viable Bacteria Non-Cultivable
WGA: Wheat Germ Agglutinin
