# Supplementary figures and images for: Cutibacterium acnes biofilm formation is influenced by bone microenvironment, implant surfaces and bacterial internalization
Source: BMC Microbiol. 2024 Jul 20;24:270. doi: 10.1186/s12866-024-03422-1 (PMC11264938; doi:10.1186/s12866-024-03422-1)

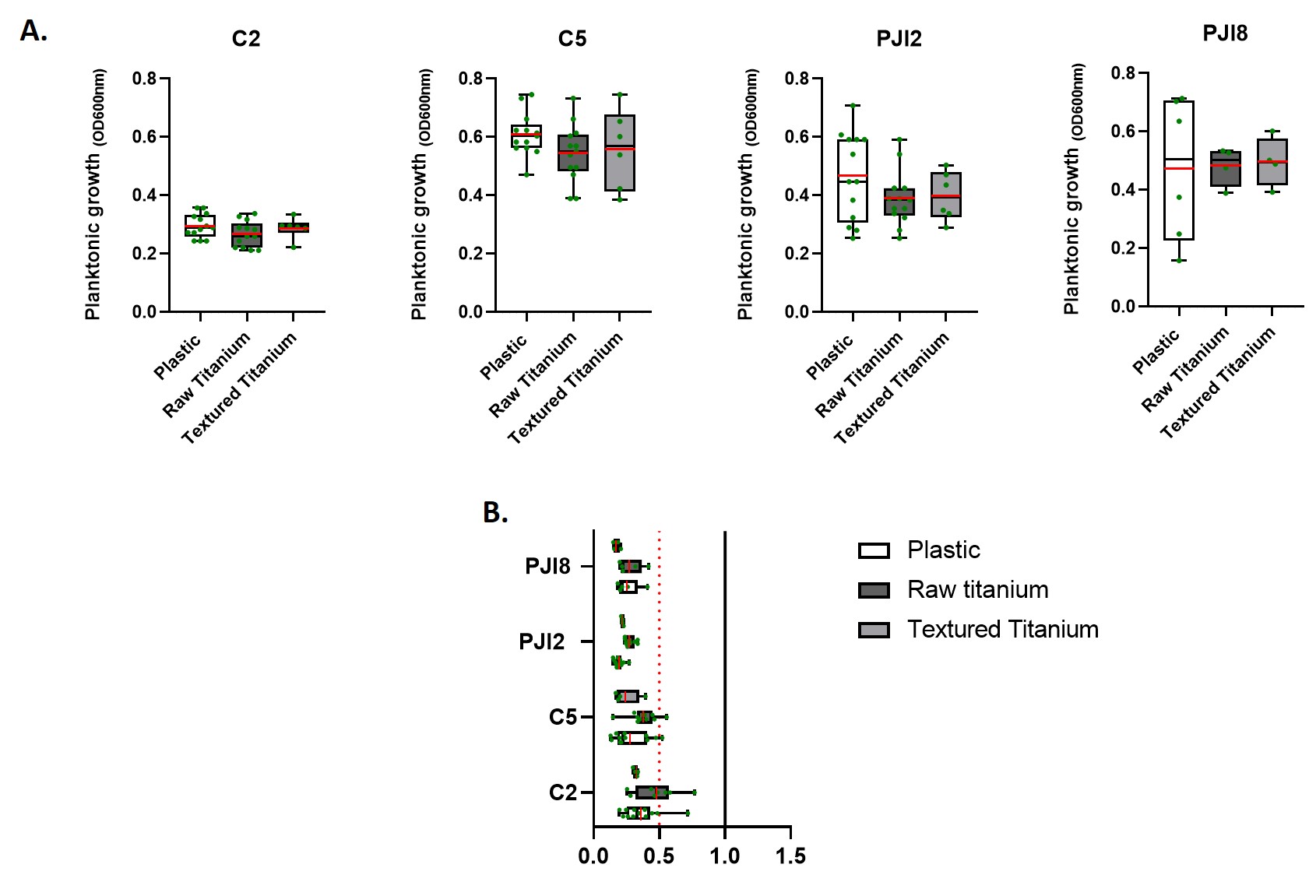

Supplement: Supplementary file 1 — Supplementary Material 1 [file 12866_2024_3422_MOESM1_ESM.jpg]

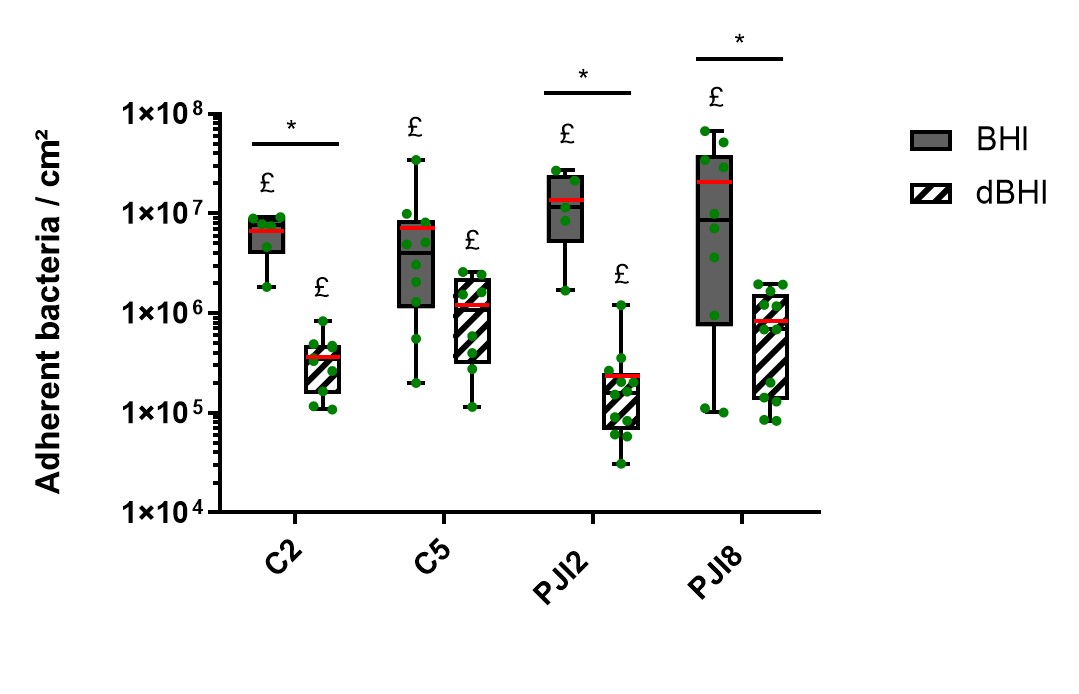

Supplement: Supplementary file 2 — Supplementary Material 2 [file 12866_2024_3422_MOESM2_ESM.jpg]

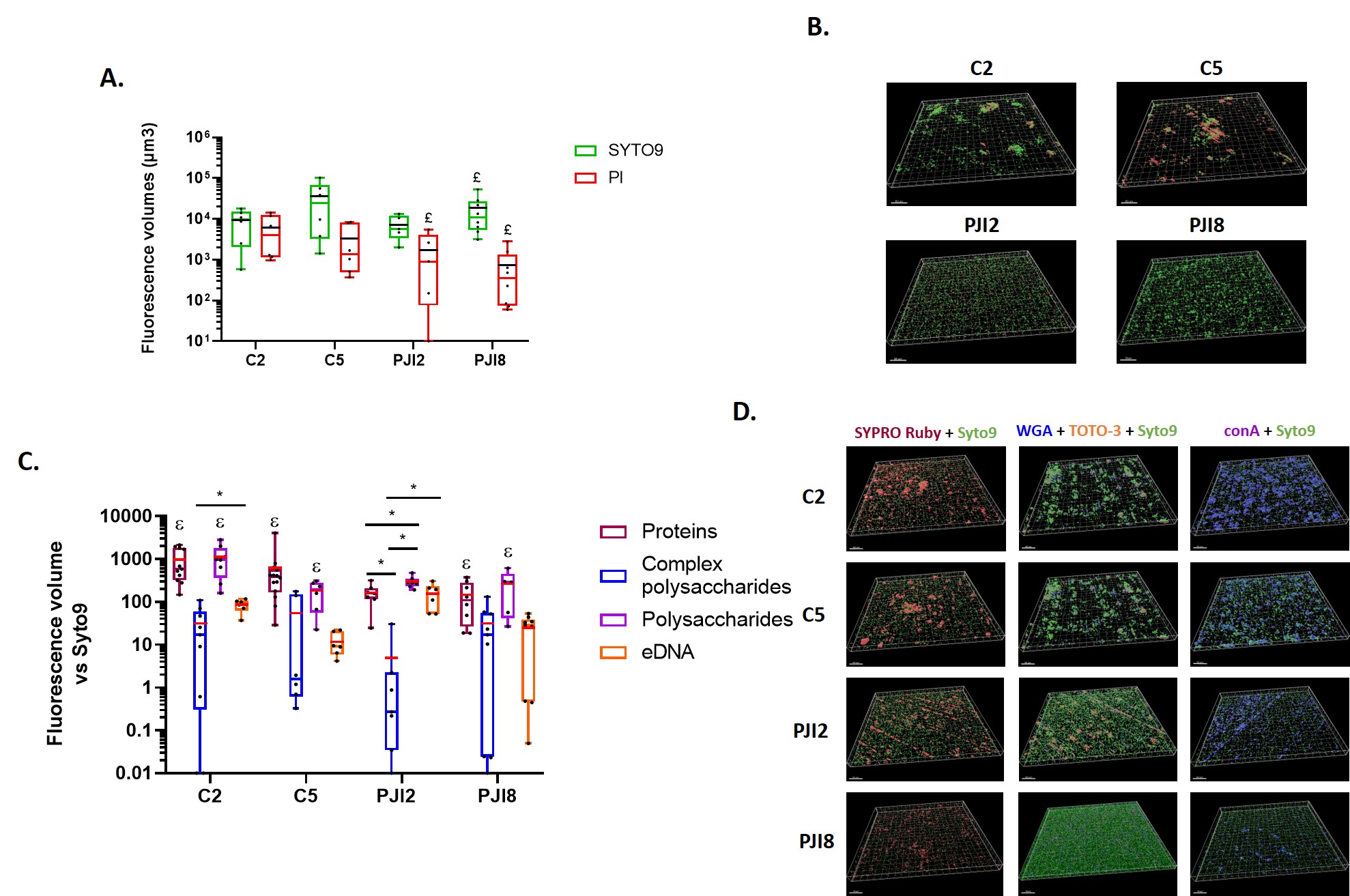

Supplement: Supplementary file 3 — Supplementary Material 3 [file 12866_2024_3422_MOESM3_ESM.jpg]

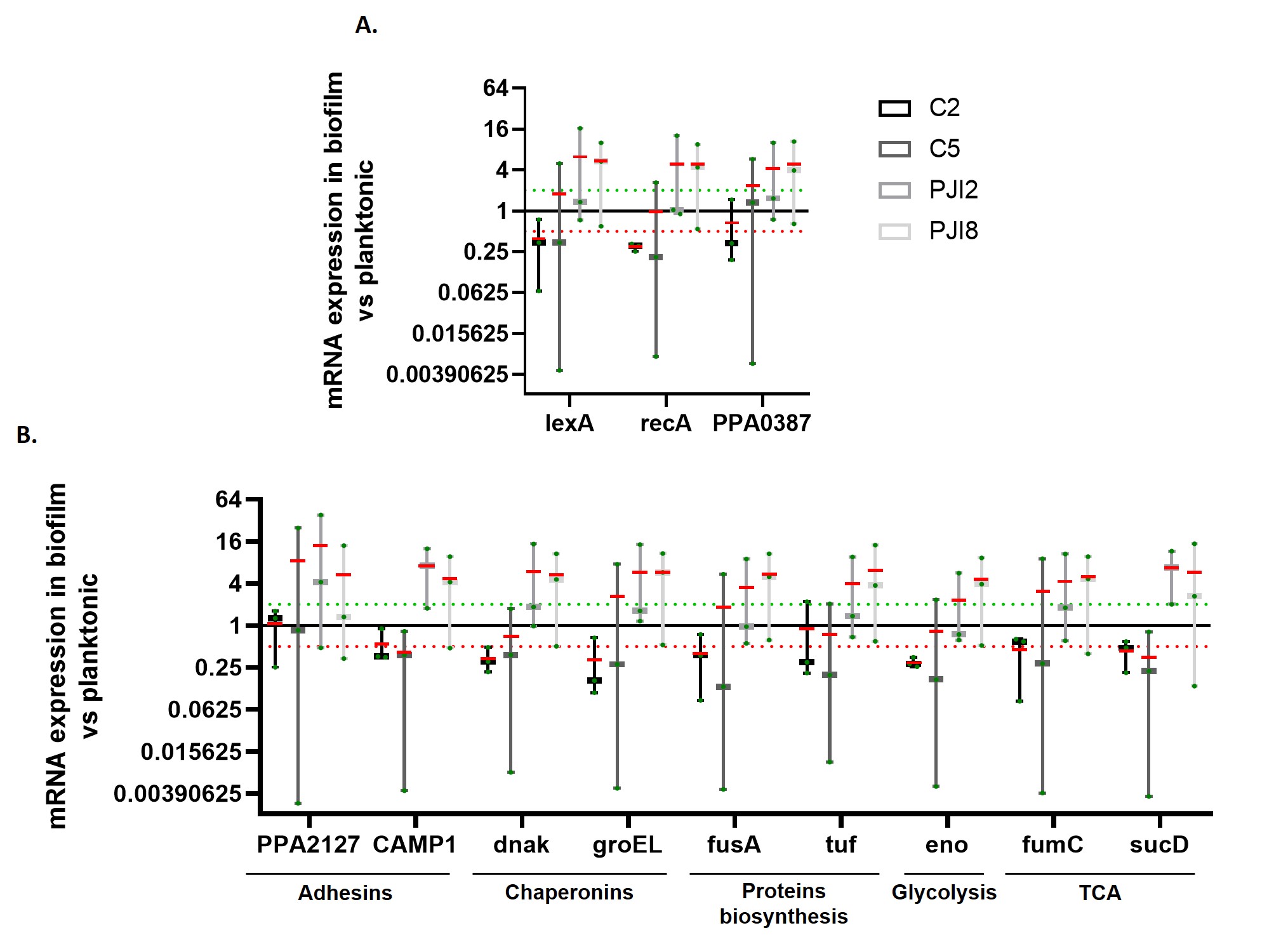

Supplement: Supplementary file 5 — Supplementary Material 5 [file 12866_2024_3422_MOESM5_ESM.jpg]

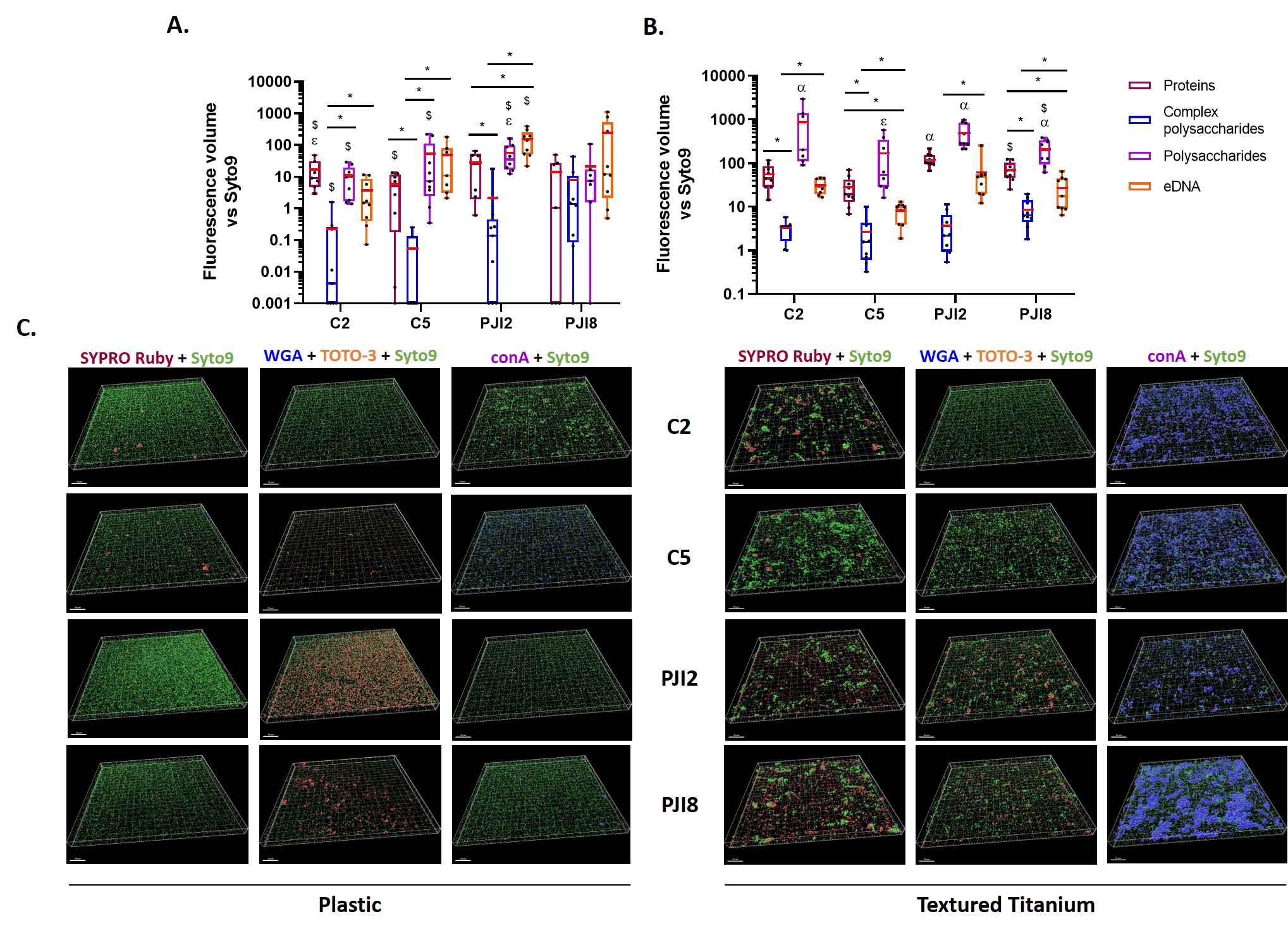

Supplement: Supplementary file 6 — Supplementary Material 6 [file 12866_2024_3422_MOESM6_ESM.jpg]
