# Supplementary material for: Cutibacterium acnes biofilm formation is influenced by bone microenvironment, implant surfaces and bacterial internalization
Source: BMC Microbiol. 2024 Jul 20;24:270. doi: 10.1186/s12866-024-03422-1 (PMC11264938; doi:10.1186/s12866-024-03422-1)
Supplement: Supplementary file 4 — Supplementary Material 4 [file 12866_2024_3422_MOESM4_ESM.docx]

**Additional files**

**Additional file 1. Nature of material does not impact bacteria planktonic growth.**

**(A)** Absorbance at 600nm of planktonic growth of *C. acnes* cultivated with BHI after incubation for 5 days. **(B)** Ratio of planktonic growth with dBHI *versus* BHI. The average value is indicated by a red bar. Experiment was performed at least 4 independent times.

**Additional file 2. *C. acnes* adheres on raw titanium support both with nutritive and diluted broth.**

Quantity of adherent bacteria cultivated with BHI (black bars) and dBHI (hatched bars) after incubation for 5 days. The average value is indicated by a red bar. Wilcoxon-Mann-Whitney test: *p*<0.05, * between BHI and dBHI and £ *versus* plastic coverslip. Experiment was performed at least 3 independent time.

**Additional file 3. *C. acnes* biofilm is mostly composed by both proteins and polysaccharides on raw titanium disk.**

**(A)** Fluorescence volumes of live and adherent bacteria, labelled by SYTO9^TM^ (green) and damaged and dead bacteria, labelled by PI (red). The average value is indicated by a black bar. **(B)** 3D-representative reconstruction of *C. acnes* biofilm morphology. **(C)** *C. acnes* biofilm composition after 5 days with dBHI. The fluorescent volumes of the matrix components: SYPRO Ruby® for proteins (dark red), Wheat Germ Agglutinin (WGA) (blue) and concanavalin A (conA) (purple) for complex and simple polysaccharides respectively, and TOTO-3^TM^ for extracellular DNA (orange) were normalized by fluorescent volumes of SYTO9^TM^ using IMARIS software after acquisitions by CLSM. The average value is indicated by a red bar. **(D)** 3D-views of matrix biofilm composition of *C. acnes*. Wilcoxon-Mann-Whitney: p<0.05, £ *versus* plastic coverslip, * between fluorescent labels and ε *versus* WGA/TOTO. Experiment was performed 3 independent times.

**Additional file 4. mRNA expression of genes is activated in PJIs-related strains biofilm on raw titanium disk*.***

**(A)** Difference of expression of stress pathway-related genes when biofilm is formed on raw titanium disk. **(B)** Levels of expression of gene involved in biofilm formation on raw titanium disks. The average value is indicated by a red bar. Experiment was performed 3 independent times.

**Additional file 5. *C. acnes* biofilm composition on textured titanium disk is mostly composed by polysaccharides after internalization.**

**(A)** *C. acnes* biofilm composition on plastic coverslip and **(B)** on textured titanium disk after 5 days with dBHI. **(C)** The fluorescent volumes of the matrix components: SYPRO Ruby® for proteins (dark red), Wheat Germ Agglutinin (WGA) (blue) and concanavalin A (conA) (purple) for complex and simple polysaccharides respectively, and TOTO-3^TM^ for extracellular DNA (orange) were normalized by fluorescent volumes of SYTO9^TM^ using IMARIS software after acquisitions by CLSM. The average value is indicated by a red bar. 3D-views of matrix biofilm composition of *C. acnes*. Wilcoxon-Mann-Whitney test, *p*<0.05: * between fluorescent labels, α *versus* each fluorochromes, ε *versus* WGA/TOTO and $ compared to non-internalized strain. Experiment was performed 3 independent times.
